# Malignant nodular fasciitis, a rare manifestation of a benign disease: case report

**DOI:** 10.3389/fonc.2026.1685315

**Published:** 2026-01-21

**Authors:** Albert J. Aboulafia, Nicole Liddy, Lauren Zeitlinger, Diana W. Molavi, Sudarsan Murali, David Aboulafia

**Affiliations:** 1 Department of Orthopaedic Surgery, The Johns Hopkins University School of Medicine, Baltimore, MD, United States; 2 Department of Orthopaedic Surgery, UPMC Children’s Specialty Services, Lancaster, PA, United States; 3 Department of Pathology, Sinai Hospital of Baltimore, Baltimore, MD, United States; 4 Department of Hematology/Oncology, Virginia Mason Medical Center, Seattle, WA, United States

**Keywords:** multifocal soft-tissue sarcoma, nodular fasciitis, soft-tissue metastasis, soft-tissue sarcoma, USP6

## Abstract

Nodular fasciitis is a common benign tumor resulting from reactive proliferation of fibroblasts and myofibroblasts. It most often presents as a firm and solitary lump in the subcutaneous fat layer or fascia adjacent to muscles in the extremities. Because of its rapid growth and high cellularity, it may mimic soft-tissue sarcoma, but it is self-limiting, often resolving with fibrosis. The evolution of benign nodular fasciitis to malignant nodular fasciitis is distinctly uncommon, and only a handful of cases have been reported in the medical literature. Herein we describe the case of a woman with a 21-year history of multifocal nodular fasciitis superimposed on malignant nodular fasciitis. Her course has been characterized by rapid tumor growth and widespread dissemination. Over the course of 2l years she underwent multiple surgical procedures. Ultimately, molecular studies demonstrated a fusion of protein phosphatase 6 regulatory subunit 3 (*PPP6R3*)–ubiquitin specific peptidase 6 (*USP6*), suggesting an oncogenic mechanism despite classic histologic features of benign nodular fasciitis with aggressive behavior. She subsequently received systemic therapy, initially with temozolomide and later pazopanib, followed by sunitinib. With the later medication she has achieved a good response of now more than 18 months. This case illustrates the importance of molecular drivers of cancer in a benign soft-tissue tumor and the potential to offer targeted therapies to extend patient survival. This rare case highlights the potential malignant behavior of nodular fasciitis associated with *PPP6R3*-*USP6* fusion.

## 1 Introduction

Nodular fasciitis is a benign, self-limiting myofibroblastic proliferation that typically presents in young adults as a rapidly growing mass, most commonly in the forearm or thigh (1–5). On imaging, it may mimic soft-tissue sarcoma, but characteristic histologic findings, including tissue-culture-like fibroblasts within a myxoid or collagenous stroma, help distinguish it from malignancy (1, 6–8). Although traditionally considered reactive, nodular fasciitis has demonstrated neoplastic potential with recent identification of ubiquitin specific peptidase 6 (*USP6)* gene rearrangements (9–11).

We report a case of deep thigh nodular fasciitis with distant metastases. Despite biopsies from multiple metastatic sites, histologic findings remained typical. Molecular analysis of the tumor yielded a unique protein phosphatase 6 regulatory subunit 3 (*PPP6R3)-USP6* gene fusion rearrangement, a novel discovery thought to account for the unusual malignant behavior. USP6 is essential in signaling pathways, functioning as a deubiquitinase leading to cellular proliferation and activation of transcription factors (12). USP6 is a deubiquitinating enzyme involved in multiple signaling pathways as a potent oncogene when overexpressed, typically through chromosomal translocations. It has been shown to drive cancer progression in mesenchymal tumors and is associated with sarcomas. It acts to remove ubiquitin chains, leading to several signaling pathways that drive cellular proliferation and transformation. Identified signaling pathways include Wnt/β-catenin, JAK/STAT, and NF-kB.

## 2 Case description

**Figure 1:**
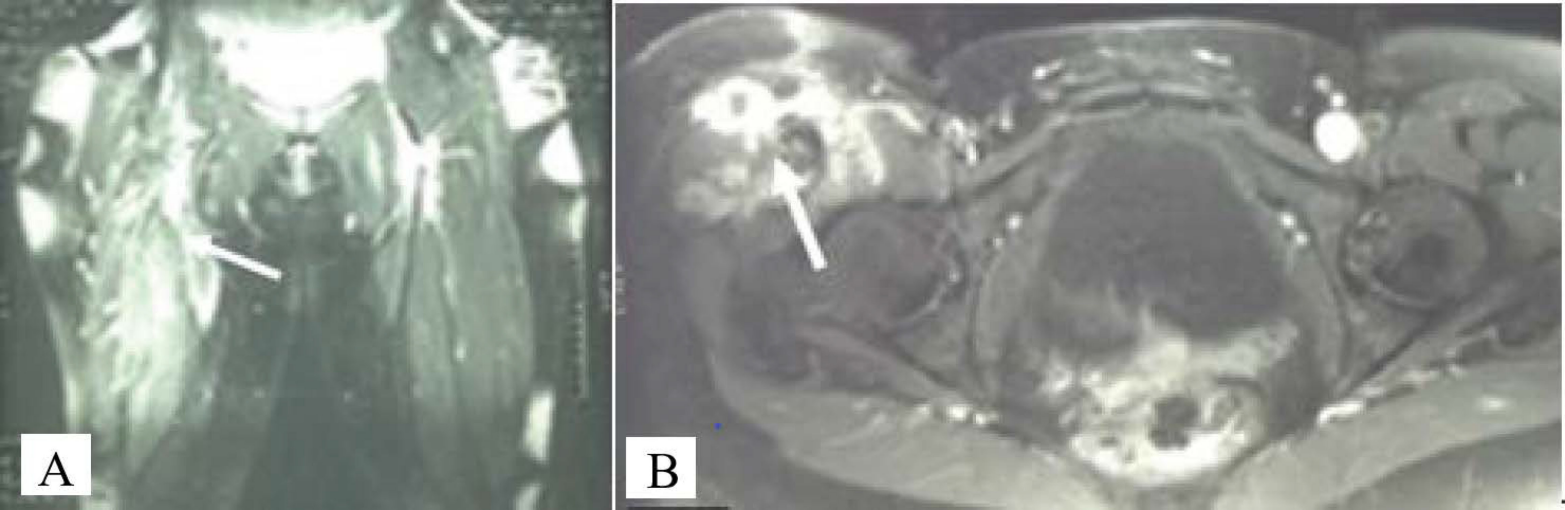
**(A)** Coronal T2-weighted magnetic resonance image of bilateral femurs taken in June 2004, with arrow showing ill-defined mass with intramuscular signal abnormalities involving the right proximal rectus femoris, vastus intermedius, and vastus lateralis muscles. **(B)** Axial T2-weighted magnetic resonance images of bilateral femurs taken in June 2005 with arrow showing progression of disease and new involvement of the sartorius, quadriceps, vastus lateralis, and iliopsoas with the tumor also now circumferentially encasing the femoral nerve.

**Figure 2:**
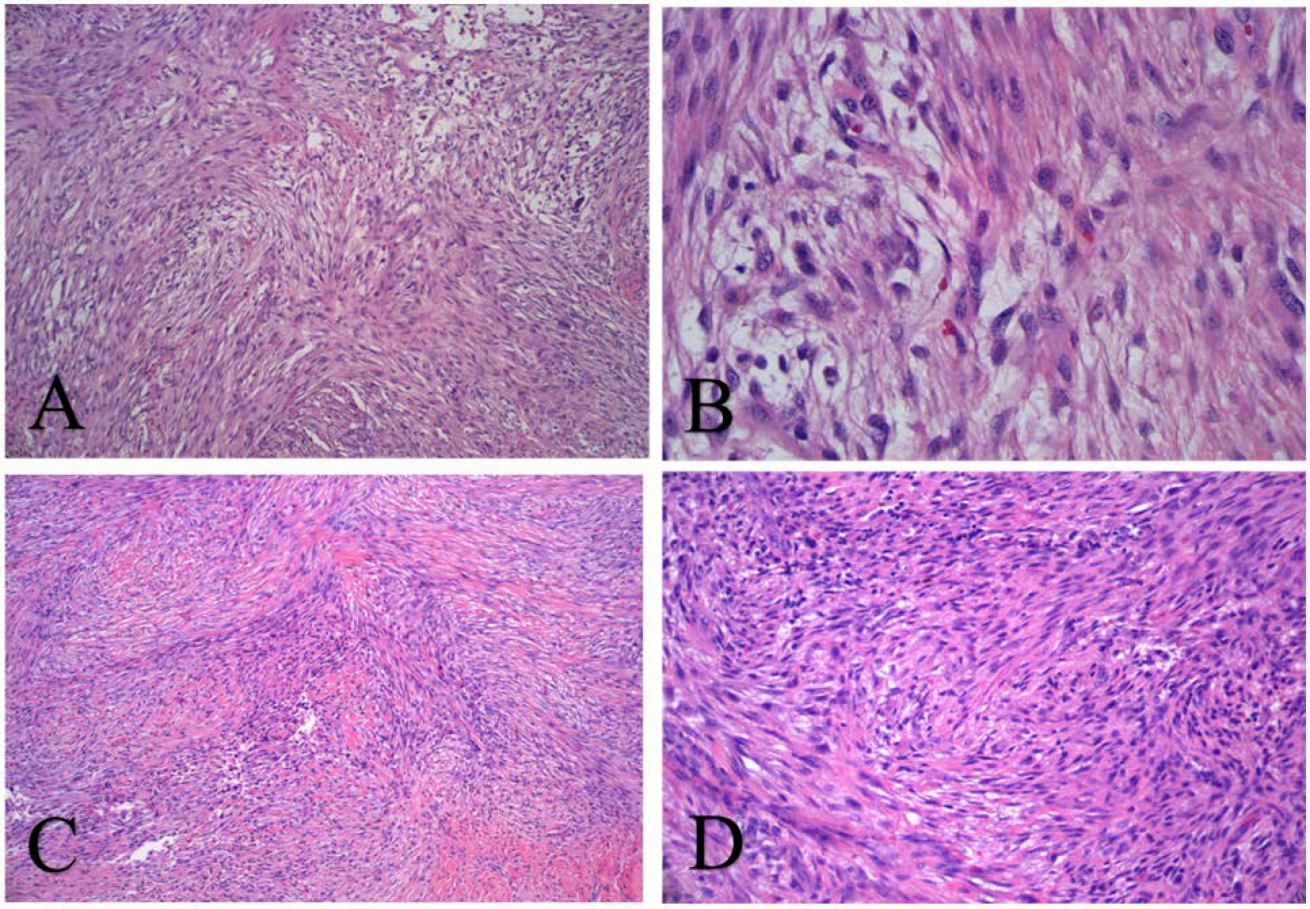
Hematoxylin and eosin staining confirming a myofibroblastic proliferation of cells in a storiform pattern on low power **(A)**, with edematous areas with extravasated red blood cells and bland, euchromatic cells with tapering processes, giving a “tissue culture” appearance on high power **(B)**, typical of nodular fasciitis. The histopathological appearance of this lesion remains unchanged over the years, including specimens obtained from the thigh **(C)** and shoulder **(D)** 13 years after diagnosis.

A 40-year-old woman with no relevant medical history presented to the musculoskeletal oncology service in 2003 with a 3-month history of right buttock discomfort after a fall. Initially treated for muscle strain, she later noticed a right groin mass with quadriceps atrophy. Examination revealed an ill-defined 10×8–cm firm, mobile, deep-seated nontender mass in the right anterolateral thigh. MRI showed an ill-defined, heterogeneous mass involving the proximal quadriceps muscles (**Figures 1A, B**). Core needle biopsy yielded a diagnosis of nodular fasciitis (**Figures 2A, B**).

Her condition remained stable until late 2004, when she developed progressive thigh pain and sensory loss. Repeat core needle biopsy reaffirmed nodular fasciitis, and she underwent intralesional resection of the tumor involving the right thigh and neurolysis of the femoral nerve, with subsequent improvement in extremity dysesthesias. Wide excision was unfeasible because of the mass’s ill-defined border and vascular encasement. Because of the persistent behavior of the tumor and lack of spontaneous regression, pathology results were reviewed by 4 soft-tissue pathologists: 3 diagnosed nodular fasciitis, and 1 suggested a “low-grade malignant process.”

Pelvic and thigh MRI 4 months later showed extensive soft-tissue changes within the anterior and medial compartments of the thigh (**Table 1**; **Figures 1C, D**), with the mass noted circumferentially around the femoral nerve. Another biopsy provided no new information. Given the lack of expected regression, an aggressive compartmental resection of the lesion and associated neurovascular structures was discussed. In the absence of a clear diagnosis of malignancy, the patient chose to continue surveillance. Inflammatory markers such as C-reactive protein and erythrocyte sedimentation rate were not collected, nor were cytokine measurements such as interleukin-6 and tumor necrosis factor-α.

**Table 1:** Chronological summary of imaging findings for a woman with malignant nodular fasciitis.

Timepoint	Imaging modality	Findings	Anatomic distribution
Early 2005	MRI	Extensive soft-tissue abnormality within the anterior and medial thigh compartments; dominant mass encasing the femoral nerve	No interval regression; biopsy nondiagnostic; surveillance chosen
2005–2011	MRI	Gradual enlargement of the primary mass; development of secondary lesions	Tumor compression contributed to deep vein thrombosis and bilateral pulmonary emboli
2013	MRI	Progressive, multifocal disease with slow enlargement; new discrete soft-tissue masses	Enlarged right anterior thigh mass (8.2×2.7×3.4 cm); new lesions in left thigh (11.3×3.4×5.4 cm), medial right thigh (12×4.8×5.1 cm), left calf (6.1×2.7×2.9 cm), left gluteus (9.1×1.2 cm), right hemipelvis (2.5×1.6 cm)
2014	FDG-PET/CT	Hypermetabolic lower-extremity lesions; additional hypermetabolic foci	SUVs 5.1–12.9; radiotherapy provided no radiographic or clinical response
Late 2018	FDG-PET/CT	Persistent, progressive, multifocal hypermetabolic soft-tissue lesions; new bilateral upper- and lower-extremity involvement	No visceral or solid-organ metastases identified
2021 (Post-pazopanib)	MRI/PET	Marked decrease in lesion size with clinical improvement	Multifocal extremity lesions
Late 2023	MRI (shoulder); FDG-PET/CT	Large infiltrative mass involving the rotator cuff musculature and joint capsule; increased metabolic activity in involved regions	Radiographic progression; transitioned to sunitinib with temporary reduction in fluorodeoxyglucose avidity

FDG-PET/CT, fluorodeoxyglucose-positron emission tomography/computed tomography; MRI, magnetic resonance imaging; SUV, standardized uptake value.

**Figure 3:**
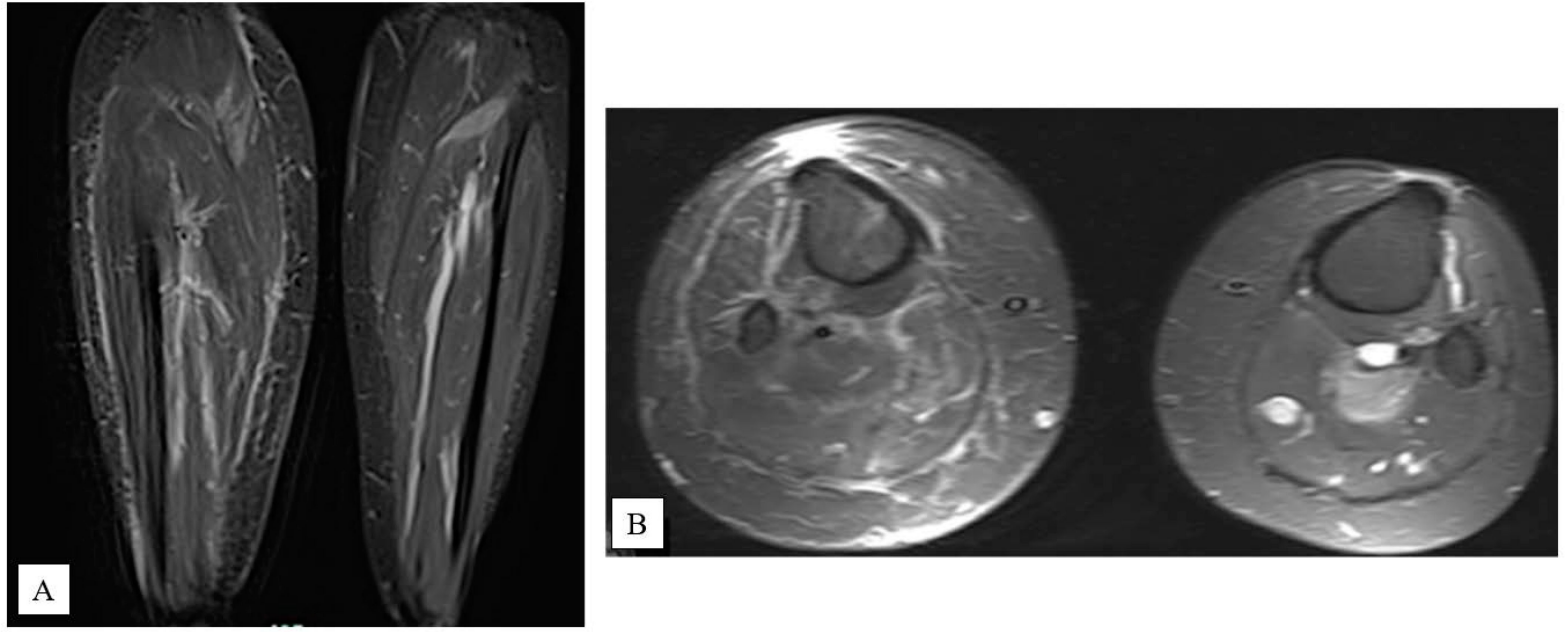
Coronal **(A)** and axial **(B)** T2-weighted magnetic resonance images of lower legs taken in December 2011. Note the right lower leg image showing a medial gastrocnemius mass measuring 1.6×2.1 cm (left arrow) and a second mass in the plantaris muscle measuring 1.9×3.1×2.3×2.6 cm (right arrow).

Between 2005–2011, the tumor grew slowly, and the patient was diagnosed with bilateral pulmonary emboli from deep vein thrombosis of the right femoral and popliteal veins due to tumor compression. MRI in 2011 showed new lesions in the medial gastrocnemius and plantaris muscles (**Figure 3**). Between 2011–2012, methylprednisolone injections provided temporary relief from calf pain and swelling. In 2013, MRI confirmed enlargement of the anterior right thigh mass (to 8.2×2.7×3.4 cm) and new masses in the left thigh (11.3×3.4×5.4 cm), medial right thigh (12×4.8×5.1 cm), left calf (6.1×2.7×2.9 cm), left gluteus (9.1×1.2 cm), and right hemipelvis (2.5×1.6 cm). Signal characteristics and several biopsies of each lesion did not vary over time, and the masses demonstrated slow, continued growth (**Table 1**).

**Figure 4:**
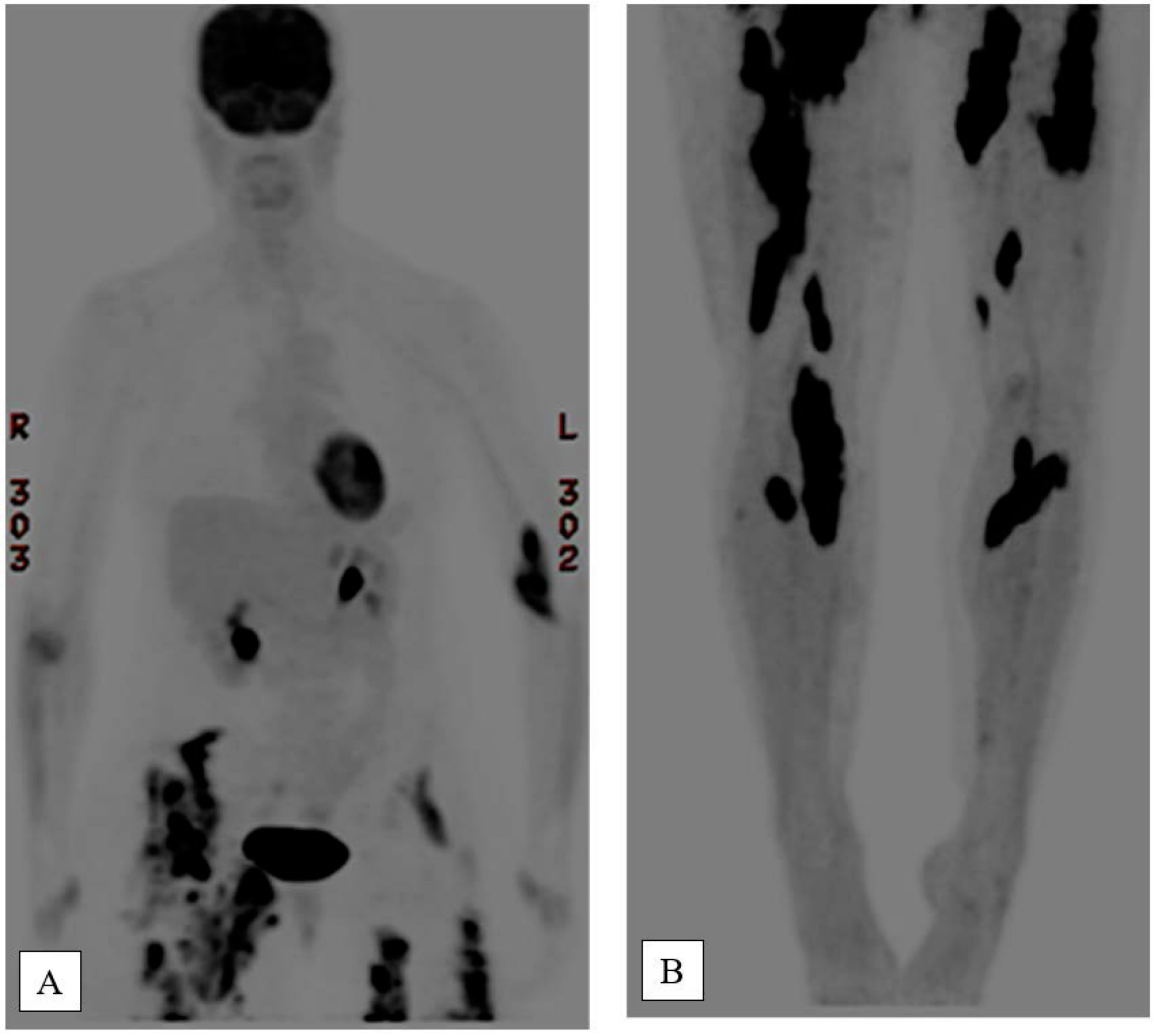
Positron emission tomography/computed tomography images of upper **(A)** and lower **(B)** body taken in late 2014, showing increased tracer uptake in the proximal right radius with maximum standardized uptake value (SUV) of 5.5; linear increased uptake in the right supinator (SUV of 3.1); left antecubital fossa (SUV of 12.0); increased tracer activity in the right lower abdomen/pelvis (SUV of 17.1); right thigh adductors (SUV of 14.1); right popliteal region (SUV of 14.4); left hip with increased uptake (SUV of 8.6); left adductors (SUV of 15.3); and left quadriceps (SUV of 13).

In 2014, the right thigh masses began to enlarge rapidly, and fluorodeoxyglucose-positron emission tomography/computed tomography (FDG-PET/CT) showed hypermetabolic activity in the lower-extremity lesions and new disease within the left antecubital fossa and right transverse abdominus musculature, with standardized uptake values ranging from 5.1–12.9 (**Figure 4**). She received a total of 50 Gy of hyper-fractionated external beam radiotherapy to areas of bulky and symptomatic disease, but this provided no relief or measurable response clinically or radiographically.

Again, aggressive sampling of the most symptomatic lesions within the right thigh, calf, and antecubital fossa showed no substantial histological change (**Figures 2C, D**), and a diagnosis of malignancy could not be rendered. However, the *PPP6R3*-*USP6* gene fusion was identified in 2016 by fluorescence *in situ* hybridization (13). On the basis of this novel gene rearrangement, she was diagnosed with malignant nodular fasciitis and received oral temozolomide (140 mg daily, 6 weeks on and 3 weeks off). This regimen was later switched to 2 weeks on therapy and 1 week off. Chemotherapy was well tolerated but provided no appreciable benefit and was discontinued.

FDG-PET/CT in late 2018 showed persistent, progressive multifocal disease with new lesions throughout bilateral upper and lower extremities and no visceral or solid-organ disease. Subsequent repeat resections yielded no pathologic change, with continued local recurrence. In mid-2021, she began systemic treatment with pazopanib 800 mg daily, which produced considerable shrinkage of the lesions. At 2 months, she had lost more than 4 kg due to improved lymphedema and was satisfied with her progress. Her disease remained stable for the next 2 years, but in late 2023, she developed a painful right posterior shoulder mass and noted progression of the left anterolateral thigh mass. PET/CT showed avid uptake in both areas, and MRI of the shoulder revealed a large, infiltrative mass involving the rotator cuff musculature and joint capsule. In response, she was transitioned from pazopanib to sunitinib, which provided temporary pain relief, and PET/CT showed decreased FDG avidity. After approximately a year, however, her symptoms began to worsen.

In 2024, she underwent resection of the left thigh and right shoulder lesions for pain control, with 70 Gy of preoperative radiation to the thigh. Since this resection, she remains on sunitinib. Pathology analysis from both sites continues to show similar malignant nodular fasciitis, and genetic tumor markers show *PPP6R3*-*USP6* fusion and an *ERBB2* (erb-b2 receptor tyrosine kinase 2) mutation (p.R188C). She is doing well, with less pain, and continued ability to walk. She continues to experience lower-extremity lymphedema and symptoms related to mass effect on adjacent neurologic structures.

## 3 Discussion

Nodular fasciitis is historically regarded as a benign, self-limiting condition (2, 5). This report builds upon the molecular findings of Guo et al. (9), providing long-term clinical follow-up, treatment outcomes, and additional genomic alterations. Guo et al. reported on the first case of *USP6*-driven oncogenesis in a case of histologically benign–appearing nodular fasciitis with malignant behavior, and the coauthors are credited with coining the term “malignant nodular fasciitis.”

Despite 21 years of histologic confirmation of benign nodular fasciitis, our patient showed progressive, multifocal disease. This atypical presentation may be explained by a novel oncogenic mechanism (*PPP6R3*-*USP6* fusion) that has been reported in other neoplasms and, more recently, in rare cases of histologically benign nodular fasciitis with clinically aggressive behavior (10, 11). Saoud et al. (10) reported a single-institution study of 7 patients with nodular fasciitis with novel *USP6* gene fusions that presented with “aggressive clinical behavior.” In 1 case over a 9-year period, the mass enlarged to the point that it created erosive changes in the adjacent femur. In another case, the patient developed pulmonary metastases and died of disease. Teramura et al. (11) reported on a patient with *PPP6R3*-*USP6* gene amplification and a mass that grew rapidly and was treated with wide resection (**Table 2**). Aside from the patient who developed pulmonary metastases, we are aware of no other cases demonstrating metastatic disease and none demonstrating soft-tissue metastases in the absence of pulmonary metastases. Thus, we reserve the term “malignant nodular fasciitis” for metastatic disease in otherwise histologically benign–appearing nodular fasciitis and not simply cases of “aggressive” local behavior.

**Table 2:** Published cases with similar presentation to the patient in the current report.

First author (year)	Gender	Age (yr)	Symptoms/Presentation	Treatment	Follow-up/outcome
Papke Jr. (2021)^a^	Male	7	Rapidly enlarging soft-tissue mass in upper extremity; histology showed striking pleomorphism beyond conventional NF features	Surgical excision 13 months after mass identification	No follow-up discussed
Tomassen (2021)^b^	Male	10	Painless swelling on right dorsal chest wall for ~1.5 weeks; firm, slightly mobile mass fixed to muscle on MRI; initially misdiagnosed as pleomorphic sarcoma on biopsy	Core needle biopsy; wide surgical excision with oncologic margins, including fascia and part of latissimus dorsi; no adjuvant therapy	Followed every ~3 months with annual CT and chest radiograph; no recurrence or metastasis at 22 months after surgery; asymptomatic
Teramura (2019)^c^	Male	27	Soft-tissue tumor with typical NF histology but aggressive, non-regressing growth and local invasion, not the usual self-limited behavior	Two open biopsies due to rapid enlargement and MRI findings; no further treatment	No follow-up discussed

CT, computed tomography; MRI, magnetic resonance imaging; NF, neurofibromatosis.

^a^Papke Jr DJ, Oliveira AM, Chou MM, Fletcher CDM. Morphologically malignant nodular fasciitis with *CALD1-USP6* fusion. *Virchows Arch* (2021) 479:1007-1012. doi: 10.1007/s00428-021-03149-8.

^b^Tomassen T, van de Ven C, Anninga J, Koelsche C, Hiemcke-Jiwa LS, Horst ST, de Leng WW, Tirode F, Karanian M, Flucke U. Nodular fasciitis with malignant morphology and a *COL6A2-USP6* fusion: a case report (of a 10-year-old boy). *Int J Surg Pathol* (2021) 29:642-647. doi:10.1177/1066896921996045.

^c^Teramura Y, Yamazaki Y, Tanaka M, Sugiura Y, Takazawa Y, Takeuchi K, Nakayama T, Kaneko T, Musha Y, Funauchi Y, et a. Case of mesenchymal tumor with the PPP6R3-USP6 fusion, possible nodular fasciitis with malignant transformation. *Pathol Int* (2019) 69:706-709. doi: 10.1111/pin.12851.

“Malignant nodular fasciitis” is a term reserved for conditions when histologic testing shows no evidence of malignancy yet the tumor behaves clinically in a malignant fashion. In this case, the patient developed extensive soft-tissue metastases (hence the term “malignant”) while histologic findings remained consistent with benign nodular fasciitis. Low-grade malignant nodular fasciitis represents a true malignant tumor. The differences lie in their disparate clinical behavior, biological features, and molecular profiles, which are essential and provide pathologists with a nuanced identification process when there is morphological overlap. Typically, nodular fasciitis grows rapidly, measures less than 5 cm, is superficial to fascia involving the upper extremities, head, neck or trunk, and (most importantly) resolves spontaneously or is cured with local excision. In contrast, low-grade malignant nodular fasciitis grows slowly, is typically larger than 5 cm, is deep to fascia, does not spontaneously resolve, and often requires wider margins to achieve local control because of its infiltrative pattern of growth.

Next-generation sequences later identified a somatic *ERBB2* p.R188C mutation in tumor tissue. Although this variant has been reported in other malignancies, including breast and lung cancer, it has not been reported in nodular fasciitis (14). This is the first reported case of nodular fasciitis with both alterations. Our patient never achieved signs of spontaneous regression nor durable response to surgery, radiotherapy, or chemotherapy. Though inexorably progressive in its clinical course, this entity also differs from the behavior of conventional soft-tissue sarcomas, showing slow local growth and extremity spread without visceral or lymphatic involvement (15).

We believe this case is the only known patient to receive chemotherapy for nodular fasciitis. Chemotherapy was initiated after identifying a *PPP6R3*-*USP6* gene fusion in her tumors. This genetic aberration results in transcriptional upregulation of *USP6*, a known oncogene in Ewing sarcoma and aneurysmal bone cysts (9, 16, 17). Molecular profiling (Caris Life Sciences, Irving, TX) was negative for the biomarkers O[6]-methylguanine-DNA methyltransferase by immunohistochemistry, suggesting a potential therapeutic effect with temozolomide. Temozolomide is an alkylating agent that has antitumor activity in gliomas and other solid tumors (18, 19). In glioma cells, it has autophagic properties that impair cellular adhesion, reducing cellular viability (20). Our patient was treated “off-label” with temozolomide because of its reliable bioavailability, relatively nontoxic effects, and broad potential for several refractory tumor types (18, 20, 21). Treatment failed to reduce tumor burden. Our patient was also trialed on tyrosine kinase inhibitor, pazopanib, with remarkable results. She was transitioned to sunitinib because of intolerable diarrhea. Sunitinib has greater tyrosine kinase activity and continues to provide excellent results while we wait for a potential clinical trial of cyclin-dependent kinase 9 inhibitor.

A previous report on nodular fasciitis describes recurrence rates of less than 2% (22). While bilateral extremity involvement has been observed, few cases of multifocal nodular fasciitis and even fewer of malignant nodular fasciitis have been reported. We present this case to highlight the malignant clinical behavior with retained benign histology and treatment-refractory nature of this disease. The major strength of this report is the 21-year longitudinal follow-up with histologic, radiologic, and molecular correlation, including response to multiple systemic therapies. However, the findings may have limited generalizability because of the uniqueness of the genetic alterations in this patient. Continued follow-up is warranted to determine whether targeted therapy against *USP6* amplification provides ongoing benefit to this patient and others with this genomic signature.

## 4 Conclusion

This case illustrates a rare and challenging presentation of histologically benign but clinically malignant nodular fasciitis, associated with a *PPP6R3-USP6* fusion and *ERBB2* mutation. Despite the lack of histologic transformation over time, the tumor exhibited progressive multifocal growth, resistance to conventional treatment, and required systemic therapy. The patient’s prolonged clinical course emphasizes the need for molecular profiling in atypical soft-tissue tumors and supports consideration of targeted therapies in future management. Furthermore, this case highlights the need for development of a molecularly guided classification system for *USP6*-driven neoplasms and supports incorporating genomic testing into the diagnostic algorithm of recurrent, multifocal/metastatic “histologically benign” myofibroblastic lesions that have a malignant clinical course.

## Data Availability

The original contributions presented in the study are included in the article/supplementary material. Further inquiries can be directed to the corresponding author.
